# Unilateral pigmented posterior lenticonus with posterior capsule defect in a pediatric patient

**DOI:** 10.1016/j.ajoc.2025.102279

**Published:** 2025-02-17

**Authors:** Jianling Yang, Geng Wang

**Affiliations:** Joint Shantou International Eye Center of Shantou University and The Chinese University of Hong Kong, Shantou, China

## 1 Claims of priority statement

After conducting a literature review on November 20, 2024 utilizing PubMed, Google Scholar, and other relevant databases, using the keywords “Pigmented Posterior Lenticonus” and “posterior capsule rupture”, we did not find any prior reports of this specific combination in the literature.

## 2 Case report

A 13-year-old boy presented with a 10-month history of progressive blurred vision in his right eye, with visual acuity reduced to 20/400 and no improvement with a −1.00-diopter correction. A school health screening five years earlier recorded right eye vision as 20/25, while the left eye vision was 20/20. There was no history of trauma, systemic diseases, or medication use.

Slit-lamp examination revealed diffuse pigmentation within the posterior lenticonus and along the surrounding posterior capsule in the right eye (Fig. A1, A2). The left eye appeared normal. Anterior segment optical coherence tomography (AS-OCT) demonstrated posterior bowing of the lens capsule with increased signal intensity (Fig. B). Scanning laser ophthalmoscopy (SLO)-fundus photography showed a normal optic disc, with macular area partially obscured by lens opacity (Fig. C1). Optical coherence tomography angiography (OCTA) revealed normal retinal vasculature, with partial obscuration by lens opacity (Fig. C2).

**Fig. A:**
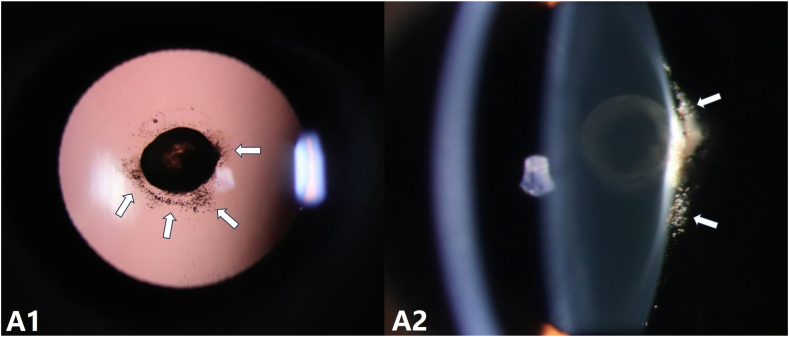
A1, A2: Slit-lamp images of the right eye using retroillumination (A1) and direct focal illumination (A2), showing diffuse pigmentation within the posterior lenticonus and along the surrounding posterior capsule (white arrow).

**Fig. B:**
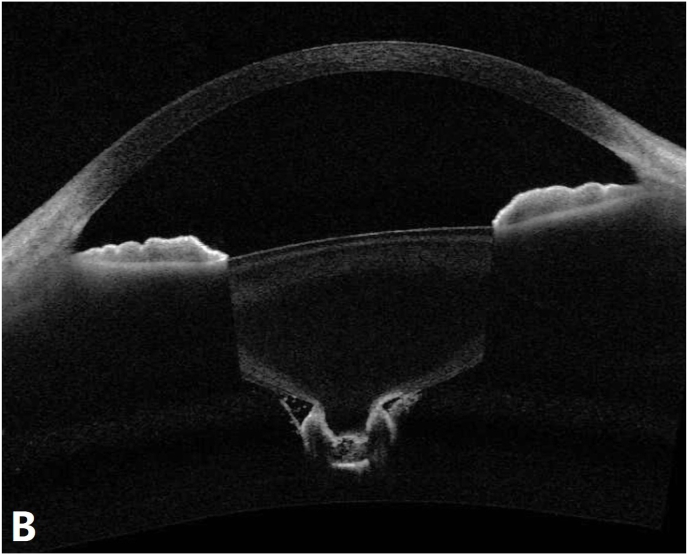
AS-OCT showing posterior bowing of the lens posterior capsule with increased signal intensity.

**Fig. C:**
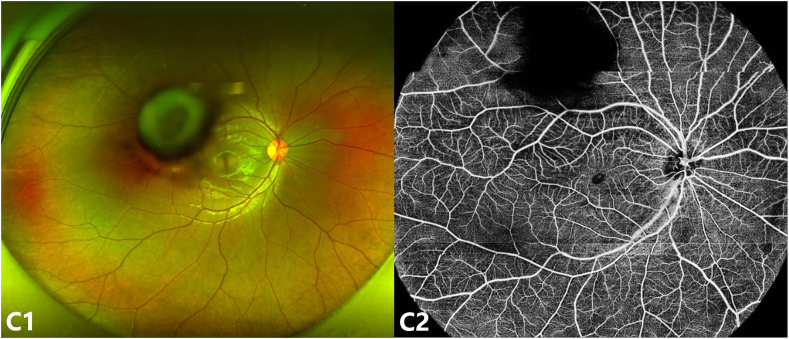
C1: SLO-fundus photography showing normal fundus with lens opacity partially obscuring the macular area. C2: OCTA showing normal retinal vasculature and part of it was obscured by lens opacity.

Phacoemulsification revealed a pre-existing posterior capsule defect (Fig. D), suggesting progressive pathology rather than a purely congenital anomaly. An anterior vitrectomy was performed, and an intraocular lens was implanted in the ciliary sulcus. At the one-month follow-up, visual acuity improved to 20/25 without complications.

**Fig. D:**
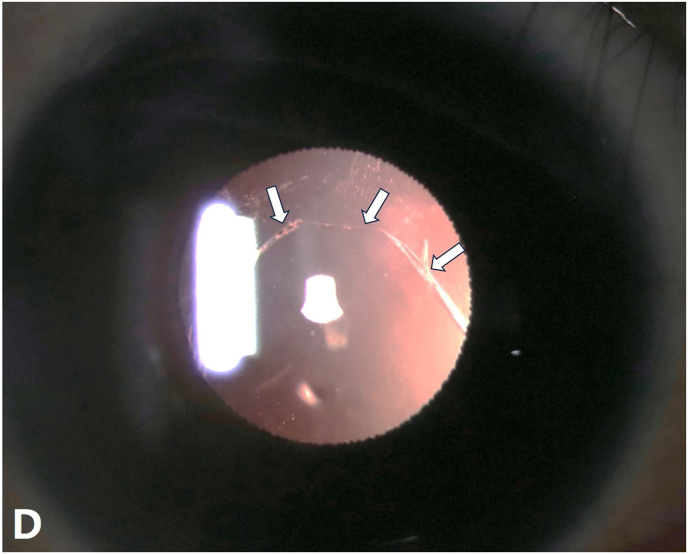
Slit-lamp image revealing a posterior capsular defect (white arrow).

## 3 Discussion

Pigmented posterior lenticonus is exceedingly rare, with only two prior cases reported.^1^^,^^2^ Previous studies attributed pigmentation to remnants of the primary hyaloid artery^1^ or the iris/ciliary body pigment epithelium.^2^ Unlike those cases, our patient exhibited posterior capsule rupture, suggesting progressive defect.

Differentiation from lens coloboma and polar cataracts is essential. Lens coloboma typically presents with equatorial notching due to zonular defects, which were absent in this case. Polar cataracts often show distinct congenital characteristics, such as concentric dense opacities at the posterior pole. While polar cataracts may occasionally involve posterior capsule rupture with localized protrusion, they rarely exhibit a conical posterior capsule configuration, as observed in this case (Fig. B). These distinctions strongly support the diagnosis of posterior lenticonus.

The progression of the defect in our patient is also noteworthy. Although his visual acuity was documented as 20/25 five years ago, the lack of detailed lens examination at that time leaves some uncertainty about the onset of the condition. Nonetheless, the morphology and clinical findings suggest a congenital defect that progressively worsened with age, rather than a newly developed anomaly.

This case underscores the importance of preoperative imaging, such as AS-OCT, in diagnosing posterior lenticonus and identifying associated capsule abnormalities. The detailed imaging and successful surgical outcome in this case provide valuable insights into the diagnosis and management of this rare condition.

## CRediT authorship contribution statement

**Jianling Yang:** Writing – original draft, Data curation, Conceptualization. **Geng Wang:** Writing – review & editing, Validation.

## Patient consent

Consent to publish this case report has been obtained from the patient's parents in writing.

## Authorship

All authors attest that they meet the current ICMJE criteria for Authorship.

## Funding

No funding or grant support.

## Declaration of competing interest

The authors declare that they have no known competing financial interests or personal relationships that could have appeared to influence the work reported in this paper.
